# Telomeres and Cancer: Resolving the Paradox

**DOI:** 10.1146/annurev-cancerbio-050420-023410

**Published:** 2021-03

**Authors:** Joe Nassour, Tobias T. Schmidt, Jan Karlseder

**Affiliations:** Molecular and Cell Biology Laboratory, The Salk Institute for Biological Studies, La Jolla, California 92037, USA;

**Keywords:** telomere, proliferative life span barriers, replicative senescence, replicative crisis, genome instability, tumorigenesis

## Abstract

Decades of study on cell cycle regulation have provided great insight into human cellular life span barriers, as well as their dysregulation during tumorigenesis. Telomeres, the extremities of linear chromosomes, perform an essential role in implementing these proliferative boundaries and preventing the propagation of potentially cancerous cells. The tumor-suppressive function of telomeres relies on their ability to initiate DNA damage signaling pathways and downstream cellular events, ranging from cell cycle perturbation to inflammation and cell death. While the tumor-suppressor role of telomeres is undoubtable, recent advances have pointed to telomeres as a major source of many of the genomic aberrations found in both early- and late-stage cancers, including the most recently discovered mutational phenomenon of chromothripsis. Telomere shortening appears as a double-edged sword that can function in opposing directions in carcinogenesis. This review focuses on the current knowledge of the dual role of telomeres in cancer and suggests a new perspective to reconcile the paradox of telomeres and their implications in cancer etiology.

## AN OVERVIEW OF HUMAN TELOMERES

The emergence of eukaryotic cells was accompanied by a transition from a circular genome to multiple linear chromosomes, enabling the propagation of multicellular organisms. Nascent linear chromosomes, however, have created many additional challenges. For instance, their DNA ends are recognized as DNA breaks and need constant shielding from DNA damage surveillance mechanisms. This task has been accomplished by the refinement of chromosome architectures and the evolution of telomeres, conserved guanine-rich terminal structures bound by specialized proteins, acting to evade and suppress most DNA damage signaling and repair pathways.

**Figure 1:**
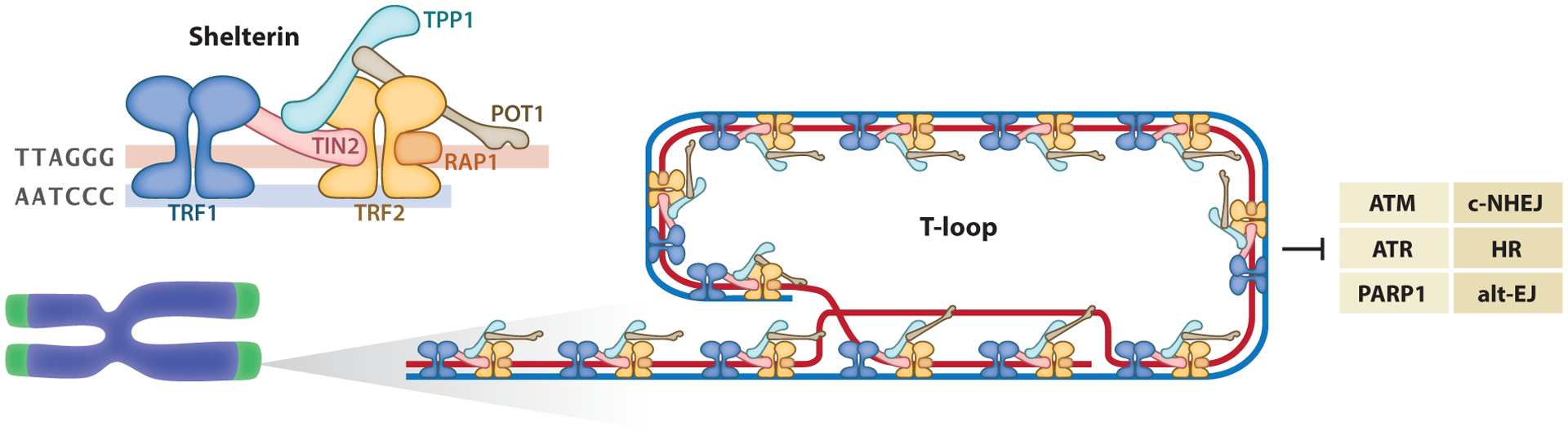
Overview of telomere composition and function. Human telomeres are formed from 4–12 kb of double-stranded TTAGGG repeats, terminating in a single-stranded 3′ overhang of a few hundred nucleotides in length. Telomeric DNA is associated with the specialized shelterin complex and remodeled into a T-loop (telomere loop) configuration. Shelterin subunits include TRF1 (telomeric repeat binding factor 1), TRF2 (telomeric repeat binding factor 2), TIN2 (TRF1-interacting nuclear factor 2), RAP1 (repressor activator protein 1), TPP1, and POT1 (protection of telomere 1). Shelterin protects chromosome ends from DNA damage signaling by ATM (ataxia telangiectasia mutated), ATR (ataxia telangiectasia and Rad3-related), and PARP1 [poly(ADP-ribose) polymerase 1] and from DNA repair by c-NHEJ (canonical nonhomologous end joining), alt-EJ (alternative end joining), and HR (homologous recombination).

In humans, telomeres comprise 4–12 kb of double-stranded TTAGGG repeats, ending in 50–400 nucleotides of single-stranded G-rich overhang (Makarov et al. 1997, McElligott & Wellinger 1997). Both single- and double-stranded fragments are embedded with an array of the six-protein complex known as shelterin (de Lange 2005) (Figure 1). The affinity of shelterin for telomeres relies on the recognition of TTAGGG repeats by three of its elements: TRF1 (telomeric repeat binding factor 1) (Broccoli et al. 1997, Chong et al. 1995), TRF2 (Bilaud et al. 1997, Broccoli et al. 1997), and POT1 (protection of telomeres protein 1) (Baumann & Cech 2001, Loayza & De Lange 2003). TRF1 and TRF2 bind the duplex part of telomeres through a C-terminal myeloblastosis domain, whereas POT1 coats the overhang with its oligonucleotide-/oligosaccharide-binding folds (Broccoli et al. 1997, Loayza & De Lange 2003). TRF1 recruits TIN2 (TRF1-interacting nuclear protein 2) (O’Connor et al. 2006), whereas TRF2 recruits RAP1 (repressor activator protein 1) (Li & de Lange 2003). Finally, POT1, TPP1, and TIN2 interact, creating a bridge linking the single- and double-stranded regions of telomeres (Houghtaling et al. 2004, O’Connor et al. 2006, Takai et al. 2011, Ye et al. 2004) (Figure 1). The assembly of shelterin does not require any posttranslational modifications of the subunits or their interactions with DNA (Erdel et al. 2017). Not all proteins present at telomeres belong to the shelterin complex. For instance, multiple telomeric proteins, such as nucleases and helicases, act as accessory factors for the shelterin complex (Palm & de Lange 2008).

Electron microscopy has revealed telomeres in the form of a closed T-loop (telomere loop) configuration, stemming from the invasion of the 3′ TTAGGG overhang into the duplex region of the same telomere (Griffith et al. 1999, Nikitina & Woodcock 2004, Raices et al. 2008) (Figure 1). Since its discovery, the T-loop has been postulated to provide protection by hiding chromosome ends from proteins that normally accumulate at DSBs (double-stranded DNA breaks). This assumption, however, has only been confirmed recently using super-resolution microcopy to visualize the structure of telomeres rendered dysfunctional by the removal of shelterin (Doksani et al. 2013, Van Ly et al. 2018). Within the shelterin complex, TRF2 serves to shape T-loops. In vitro, TRF2 is capable of remodeling telomeric substrates into looped structures, suggesting that TRF2 has some inherent ability to change the structure of telomeric DNA (Stansel et al. 2001). This feature depends on the TRF homology domain, which is capable of wrapping ~90 bp (base pairs) of DNA around itself (Amiard et al. 2007, Poulet et al. 2009). Further, TRF2 can bind and protect Holliday junctions, DNA structures resembling those present at the base of T-loops (Fouché et al. 2006, Nora et al. 2010, Poulet et al. 2009). Finally, TRF2 deletion in murine and human cells results in a rapid destabilization of T-loops and linearization of telomeres (Doksani et al. 2013, Van Ly et al. 2018).

Shelterin prevents chromosome termini from activating a network of signaling cascades known as the DNA damage response (DDR) (Palm & de Lange 2008). This function was revealed by a series of studies where individual or multiple shelterin components were deleted in mouse and human models (Sfeir & de Lange 2012). In general, the removal of shelterin components results in phenotypes where the chromosome ends evoke either an ATM-, ATR-, or PARP1-dependent DDR and the telomeres are subjected to homologous recombination (HR) or fusion via canonical nonhomologous end joining (c-NHEJ) or alternative end joining (alt-EJ) (Rai et al. 2010, Smogorzewska et al. 2002) (Figure 1). The current state of knowledge of how shelterin prevents these signaling pathways suggests that TRF2 suppresses the ATM-dependent response; TPP1 and POT1 suppress the ATR response; TRF2 and RAP1 inhibit c-NHEJ, TRF2, TPP1, and POT1; Ku70 and Ku80 prevent alt-EJ; and RAP1, POT1, Ku70, and Ku80 protect from HR, all of which have been reviewed in detail by de Lange (2018).

## TELOMERE-BASED PROLIFERATIVE LIFE SPAN BARRIERS

Over 40 years ago, the Soviet scientist Alexei Olovnikov and American scientist James Watson recognized a fundamental problem linked to the replication of linear DNA molecules (Olovnikov 1973, Watson 1972). Both scientists came to the conclusion that the replication of linear DNA is troublesome, as it must be accompanied by a loss of terminal DNA. How eukaryotic cells managed to solve this end-replication problem became a central question that eventually connected telomeres to replicative aging.

### The Three-Stage Model of Replicative Aging

**Figure 2:**
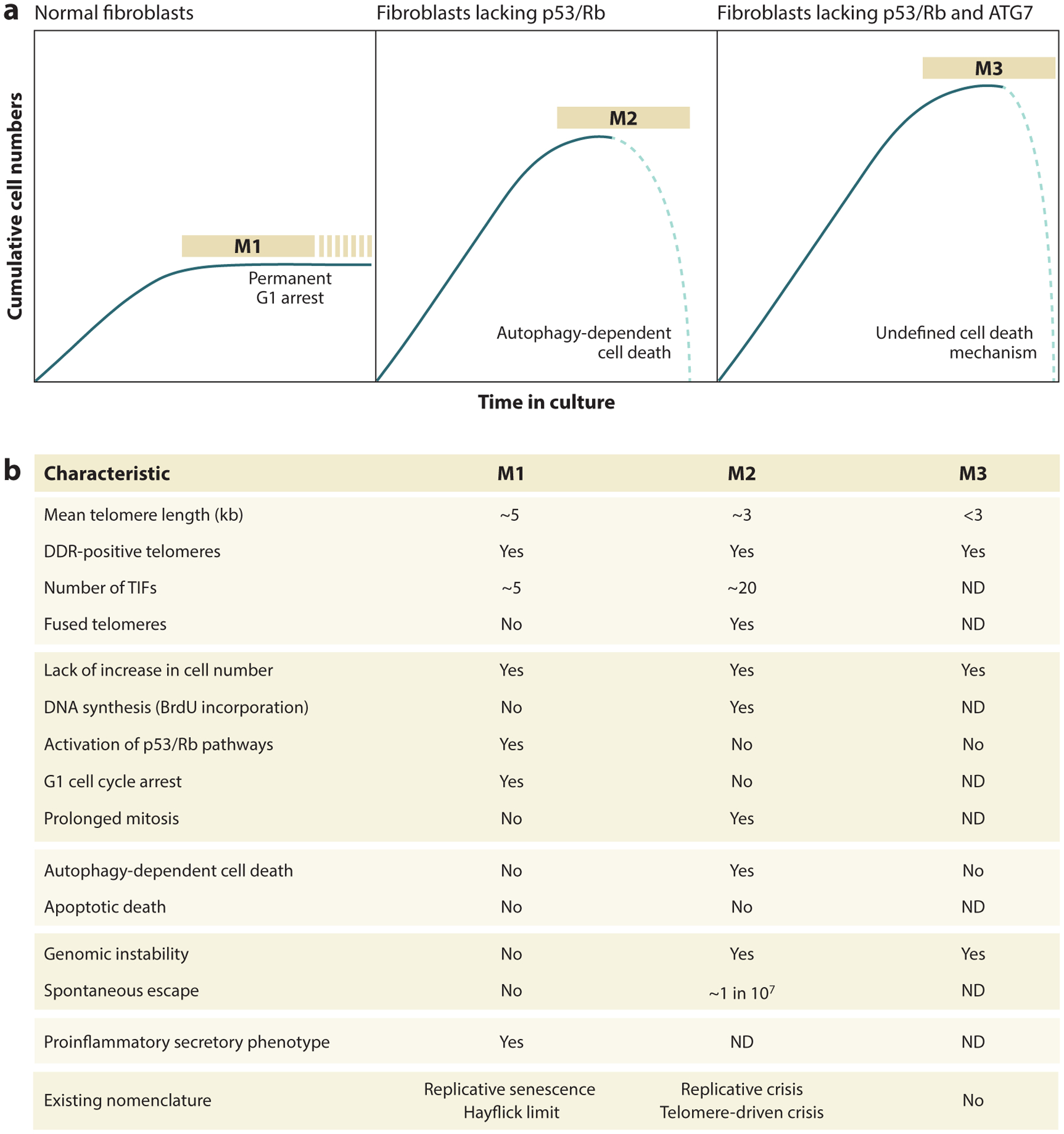
Telomere-based proliferative barriers. Telomere shortening imposes three proliferative barriers on dividing cells: senescence (M1), crisis (M2), and M3. Replicative senescence is a stable form of cell cycle arrest induced as a primary response to shortened telomeres and activation of a signaling network known as DDR. Suppression of p53 and Rb pathways causes a significant extension of in vitro life span. However, these cultures eventually also cease dividing and enter M2, during which extremely short telomeres become subject to DNA repair activities, leading to chromosome fusions and widespread cell death. Loss of either autophagy or cGAS-STING allows cells to proliferate beyond M2 limits and enter a third proliferative barrier. This end point has been designated M3. (*a*) Growth curves of normal human fibroblasts, fibroblasts lacking the p53/Rb pathway, and fibroblasts lacking both the p53/Rb and autophagy pathways. (*b*) Summary of human fibroblast characteristics at different growth plateaus. Abbreviations: BrdU, 5-bromo-2′-deoxyuridine; DDR, DNA damage response; M1/2/3, mortality stage 1/2/3; ND, not determined; Rb, retinoblastoma protein; TIFs, telomere dysfunction–induced foci.

In contrast to cancer cell lines, human somatic cells derived from healthy tissues lack TMMs (telomere maintenance mechanisms) and their telomeres shorten at a rate of 50–150 bp with each round of DNA replication (Harley et al. 1990). Accordingly, their growth is limited to a certain number of population doublings and comprise a near-exponential growth phase followed by a phase of stagnation (plateau) marked by the absence of net growth (Counter et al. 1992, Harley et al. 1992, Hayflick 1965, Wright & Shay 1992) (Figure 2a). The timing of onset of this growth plateau is for the most part dictated by the lengths of telomeres, which in turn influence the integrity of shelterin units and the stability of T-loops. In growing cells, telomeres are usually present in a protected closed state, where chromosome termini are shielded against DNA damage surveillance mechanisms (de Lange 2005). After a certain number of cell divisions, however, telomeres become too short to adopt a looped conformation, resulting in their linearization and the exposure of chromosome termini as substrates to the DDR pathway (d’Adda di Fagagna et al. 2003, Herbig et al. 2004, Kaul et al. 2012).

While linear telomeres are generally sensed by DDR factors, a considerable fraction could remain resistant to c-NHEJ-mediated fusions (Cesare et al. 2013, Hayashi et al. 2012). This has led to the concept that linear telomeres could exist in two distinct states: (*a*) an intermediate state that is partly protective, as telomeres enable DDR signaling but can sufficiently bind shelterin to suppress fusions, and (*b*) an uncapped state, where telomeres are both DDR positive and fusogenic, potentially resulting from a level of shelterin binding that is insufficient to suppress DNA repair machineries (Cesare & Karlseder 2012). Linear telomeres have the potential to elicit cell cycle arrest and cell death programs that counterbalance cell proliferation, resulting in growth plateaus known to function as potent barriers to malignant transformation (Wright & Shay 1995).

Telomere shortening implements two distinct proliferative boundaries on dividing primary fibroblast and epithelial cells: senescence [mortality stage 1 (M1)] and crisis [mortality stage 2 (M2)] (Wright & Shay 1992) (Figure 2a). Senescence is activated as a primary response to telomere shortening, when one or a few telomeres become identified as broken DNA ends and induce a network of DNA damage signaling that prevents additional cell divisions (d’Adda di Fagagna et al. 2003, Herbig et al. 2004, Takai et al. 2003). Suppression of p53 and Rb (retinoblastoma protein) pathways, e.g., through expression of certain viral oncogenes such as human papilloma virus E6 and E7 proteins, renders cells insensitive to DDR signals emanating from telomeres and incapable of exiting the cell cycle. As a result, they continue to divide and their telomeres shorten beyond the point that would normally occur in senescence. These cultures, however, are not immortal, as they eventually succumb to a second proliferative barrier, often referred to as crisis, accompanied by a nearly complete loss of viable cells. Mean telomeric lengths reported for cells in crisis are very low (2–3 kb), suggesting that some chromosome ends may have been eroded into sub-telomeric regions (Capper et al. 2007). Telomeres that have shortened to this degree are almost completely denuded of telomeric repeats and can no longer bind shelterin proteins, characteristics of uncapped-state telomeres. Thus, telomeres become subject to DNA repair activities, resulting in their fusion with other telomeric loci or nontelomeric DSBs, and chromosomal aberrations such as micronuclei (MN) and chromatin bridges emerge (Davoli & de Lange 2012, Maciejowski et al. 2015). Cells in replicative crisis frequently undergo cell death. A functional link between autophagy and the DNA-sensing pathway has been discovered recently, in which the antiviral cGAS (cyclic GMP-AMP synthase)-STING (stimulator of interferon genes) pathway triggers an atypical form of autophagy that, instead of supporting recycling of cellular components, causes cell death in the vast majority of the population (Nassour et al. 2019). Crisis can be viewed as a backup tumor-suppressor mechanism for replicative senescence, during which p53-negative cells with uncapped-state telomeres are removed by autophagy-dependent cell death (Figure 2a).

Senescence and crisis are two independent cellular programs, induced by qualitatively distinct telomere states, defined by contrasting features and, most importantly, associated with different biological outcomes (Figure 2). The current literature lacks a clear definition of replicative crisis, and there continues to be confusion regarding this cellular program. What causes replicative crisis and what defines a cell in crisis are fundamental questions that have not been addressed in depth. In the following section, we propose a description of cellular crisis and highlight the main distinctions with senescence (Figure 2).

### Replicative Senescence: Causes and Characteristics

Senescence has long been seen as a powerful tumor-suppressive mechanism that prevents cells with extensive DNA damage or a very active stress signaling response from becoming immortal. Many triggers can induce senescence, such as oncogene activation, DSBs, oxidative stress, nutrient depletion, epigenetic changes, and, of course, telomere dysfunction. Here, we focus on replicative senescence, which is triggered by replication-associated telomere shortening.

#### Intermediate-state telomeres.

Replicative senescence is induced by intermediate-state telomeres capable of initiating DDR signaling pathways with no concomitant fusions. It was initially reported that senescence coincides with the disappearance of the G-rich 3′-ended telomeric overhang and that it is the global loss of this overhang that initiates senescence (Stewart et al. 2003). Parallel studies, however, have argued that senescence results from a change in the protected status of a few shortened telomeres rather than from a complete loss of telomere protection through overhang erosion (Hemann et al. 2001, Karlseder et al. 2002). Telomere length profiles in senescent cells are quite heterogeneous and range from 7 to 4 kb, indicating that not all telomeres contribute to the onset of senescence (Harley et al. 1990). Further, senescent cells exhibit a small number of DDR-positive telomeres, indicating that only a subset of short telomeres trigger the growth arrest (d’Adda di Fagagna et al. 2003, Kaul et al. 2012). This is in accordance with the findings that a single DSB is sufficient to induce cell cycle arrest (Deckbar et al. 2007, Löbrich & Jeggo 2007).

#### G1 growth arrest.

An essential feature of replicative senescence is an irreversible cell cycle arrest, usually in G1 phase, through the activation of two major tumor-suppressor pathways, the p53/p21^Waf1/Cip1^ and p16^Ink4a^/Rb pathways (Ben-Porath & Weinberg 2005). In contrast to mouse cells, prevention of senescence in human fibroblasts requires the suppression of both p53 and Rb, indicating that both pathways are activated in parallel and possess redundant functions (Shay et al. 1991, Smogorzewska & de Lange 2002). The involvement of p16^Ink4a^ in replicative senescence has been controversial. Telomere shortening in primary cells was shown to induce p16^Ink4a^ (Alcorta et al. 1996, Hara et al. 1996), and maintenance of telomeres by telomerase abolished p16^Ink4a^ upregulation (Bodnar et al. 1998). Accordingly, expression of the TRF2 dominant-negative allele TRF2^ΔBΔM^ was shown to provoke a p16^Ink4a^-dependent growth arrest (Smogorzewska & de Lange 2002). However, p16^Ink4a^ deficiency in these cells only partially restored the growth arrest, suggesting that p16^Ink4a^ may act as a secondary mechanism maintaining the growth arrest (Jacobs & de Lange 2004).

#### Resistance to cell death.

Senescent cells are viable, metabolically active, and resistant to certain apoptotic signals. The last of these attributes might explain why senescent cells tend to accumulate in several tissues with aging. The mechanisms by which senescent cells resist apoptosis are not fully understood and could involve an upregulation of antiapoptotic proteins of the BCL-2 protein family, such as BCL-W and BCL-XL, since silencing of these proteins was shown to be successful in inducing apoptosis in senescent cells (Yosef et al. 2016).

#### cGAS-STING proinflammatory pathway.

Senescent cells experience significant metabolic changes and produce a wide spectrum of immune and inflammatory mediators, collectively termed senescence-associated secretory phenotype (SASP) (Coppé et al. 2006). The SASP performs two conflicting roles in tumorigenesis. On the one hand, it stimulates the growth of neighboring pretransformed cells (Coppé et al. 2010, Krtolica et al. 2001), and on the other hand, it participates in the clearance of senescent cells by attracting immune cells via a process called senescence surveillance (Kang et al. 2011). The antiviral cGAS-STING signaling pathway has recently been identified as a SASP regulator (Glück et al. 2017, Yang et al. 2017). Multiple nonexclusive mechanisms for cGAS-STING activation during senescence have been proposed. First, downregulation of Lamin B1 leads to alterations in the nuclear envelope and release of cytoplasmic chromatin fragments into the cytosol (Dou et al. 2017). Second, downregulation of the cytoplasmic DNases, DNase2 and TREX1, results in accumulation of cytosolic DNA, promoting SASP in senescent cells (Takahashi et al. 2018). Finally, increased LINE-1 (long-interspersed element 1) transcription and reverse transcription during senescence facilitate cytoplasmic DNA accumulation and trigger cGAS-STING-dependent SASP (De Cecco et al. 2019).

#### Genome stability.

Senescence is not accompanied by gross chromosomal abnormalities (Romanov et al. 2001, Walen & Stampfer 1989). Cytogenetic analysis of senescent cells has revealed the absence of structural and numerical chromosomal abnormalities: No translocations, deletions, or other rearrangements (i.e., telomere fusions) were detected in senescent cells.

### Replicative Crisis: Causes and Characteristics

Crisis is a fascinating mechanism that serves to clear cells from the population that have bypassed or escaped replicative senescence. Since such cells have lost p53- and Rb-dependent tumor suppressors, crisis had to evolve p53-/Rb-independent pathways to trigger cell death. Here, we focus on recent developments that address mechanisms of cell death in replicative crisis.

#### Uncapped-state telomeres.

Following the inactivation of cell cycle checkpoints, senescence-bypassed cells reach a second proliferative barrier known as crisis, during which critically short telomeres can no longer bind shelterin proteins and become vulnerable to fusions with other telomeres or nontelomeric DSBs. The pattern of accumulation of fused chromosomes suggests that crisis involves both qualitative changes from the senescence state (intermediate state in senescence versus uncapped state in crisis) and quantitative changes (few linear telomeres in senescence versus many in crisis). Fusion of telomeres in crisis is often associated with deletions and microhomology at the fusion site, which are characteristic of alt-EJ rather than c-NHEJ, which is independent of resection (Capper et al. 2007, Jones et al. 2014, Letsolo et al. 2010). However, telomeres lacking TRF2 are normally repaired through c-NHEJ (Smogorzewska et al. 2002). Therefore, short telomeres in crisis seem to be processed differently, a situation exemplified in telomerase-deficient mice, where fusion of short telomeres occurs independently of c-NHEJ (Maser et al. 2007, Rai et al. 2010). The reason for this difference is not yet clear.

#### Prolonged mitosis.

In contrast to senescence, cells undergoing replicative crisis are not permanently arrested in interphase. Instead they suffer from delayed mitotic progressions that can last up to several hours (Hayashi et al. 2015). This so-called prolonged mitosis phenotype involves a sustained activation of the spindle assembly checkpoint, leading to cell death during the same mitosis or next interphase. In fibroblasts, dicentric chromosomes formed by telomere-telomere fusions are responsible for mitotic arrest and cell death (Hayashi et al. 2015). Neither arrest nor cell death was observed in epithelial cells, in which dicentric chromosomes persist during cytokinesis and evolve into extended chromatin bridges (Maciejowski et al. 2015). As the daughter chromosomes migrate away from one another, the DNA bridge becomes attacked by the cytoplasmic 3′ exonuclease TREX1, leading to resolution of the conjoining sequence and generation of free ends that may initiate BFB (breakage-fusion-bridge) cycles or chromothripsis (Maciejowski et al. 2015). Thus, the behavior of epithelial cells in response to fused telomeres differs from that of fibroblasts. These observations could provide a molecular explanation for the higher risk of malignancy stemming from epithelial versus mesenchymal tissue.

#### Autophagy-dependent cell death.

The mechanism by which crisis cells die has remained elusive until very recently. It has generally been assumed that apoptosis is the major mode of cell death in crisis; however, that assumption had never been formally proven. In a recent study, we undertook a detailed characterization of cell death mechanisms occurring in crisis and discovered a key role for macroautophagy (hereafter referred to as autophagy) (Nassour et al. 2019). Cells transiting through crisis displayed biochemical and morphological features of ongoing autophagy, including an increased expression of autophagy-related proteins, accumulation of autophagic vacuoles (autophagosomes and autolysosomes), and enhanced autophagic flux (Nassour et al. 2019). However, markers of apoptosis-like cell death were undetectable. Thus, autophagy plays an essential role in facilitating cell death during crisis in both epithelial and fibroblast cells. Indeed, cells lacking autophagy-related genes (*ATG3*, *5*, and *7*) resisted cell death, continued to proliferate, and bypassed the crisis plateau (Nassour et al. 2019). Crisis bypass upon loss of autophagy was associated with a significant accumulation of chromosomal aberrations and emergence of cells with cancer-relevant genome alterations. Spectral karyotyping analysis revealed extensive chromosomal aberrations, including nonreciprocal translocations, deletions, and aneuploidy (Nassour et al. 2019). Collectively, these findings established a novel role of autophagy in the clearance of cells during crisis, thereby restricting the propagation of cells with unstable genomes.

The role for autophagy in suppressing tumor initiation is further supported by mouse genetic studies showing a considerable increase of tumor frequency when autophagy is impaired (Grishchuk et al. 2011, Qu et al. 2003, Rao et al. 2014, Takamura et al. 2011). It is worthy to note, however, that the role of autophagy in cancer is dynamic and depends, in part, on tumor type and stage. While autophagy prevents cancer initiation, it promotes growth and survival of established cancers (White & DiPaola 2009).

#### cGAS-STING prodeath pathway.

The ability of critically short telomeres to induce autophagy-dependent cell death during crisis relies on the release of nuclear DNA into the cytosol and activation of the cGAS-STING DNA-sensing pathway (Nassour et al. 2019). Crisis cells display high levels of cytosolic DNA species, including MN with ruptured nuclear envelopes. MN are highly positive for the DNA sensor cGAS, as well as the autophagosomal protein LC3, highlighting a cross talk between the intracellular DNA-sensing pathway and autophagy machinery.

There are still many unanswered questions about how critically short telomeres drive the formation of cytosolic DNA species. MN are frequent in cells experiencing telomere fusions, yet the underlying mechanisms are not defined. Previous observations have indicated that dicentric chromosomes, products of telomere fusions, contribute to the nuclear envelope breakdown via a process called NERDI (nuclear envelope rupture during interphase) (Maciejowski et al. 2015). Such events might lead to the formation of MN through two distinct mechanisms: (*a*) nuclear herniation of large amounts of chromatin from the nucleus, and/or (*b*) entry of harmful cytoplasmic components (endo- or exonucleases) into the nuclear space and formation of acentric fragments. Further studies are needed to uncover the underlying mechanisms.

Upon loss of the cGAS-STING pathway, cells evade autophagy-dependent cell death and continue to divide past the crisis barrier (Nassour et al. 2019). Such cells eventually reach a third proliferative block, culminating in a period of massive cell death. Based on the original nomenclatures M1 and M2, we propose the term “mortality stage 3 (M3)” to designate the third plateau (Figure 2). Cells transiting through M3 are fundamentally different from crisis, despite the fact that they both undergo cell death. Loss of viability at the third plateau might be driven by chromosome breakage, although it may also involve additional mechanisms. Future research will clarify this point.

#### Genome instability.

Telomere fusions are a major source of genomic alterations found among different tumors. These include amplifications, loss of heterozygosity, and translocations through BFB cycles (Artandi et al. 2000, Riboni et al. 1997, Roger et al. 2013). The repertoire of telomere-driven genomic aberrations has been recently extended to comprise chromothripsis, a new type of complex chromosomal rearrangement detected in multiple cancers and some congenital disorders (Li et al. 2014, Maciejowski et al. 2015, Mardin et al. 2015). Chromosomes experiencing chromothripsis first shatter into several fragments and then get ligated back together in a random manner, most likely through c-NHEJ. This creates highly rearranged chromosomes from a single catastrophic event (Stephens et al. 2011). Although it was initially considered a relatively rare event in the development of cancers, it is now well accepted that chromothripsis represents a widespread mutational phenomenon found in diverse tumor types (Cortés-Ciriano et al. 2020).

A recent study has provided a mechanistic explanation for how short telomeres during crisis could lead to chromothripsis (Maciejowski et al. 2015). The authors employed live-cell imaging and monitored the fate of dicentric chromosomes formed upon the removal of TRF2 in p53-/Rb-deficient epithelial cells. Dicentric chromosomes developed into long bridges surrounded by altered nuclear envelopes and such DNA bridges were subsequently resolved by the cytoplasmic exonuclease TREX1. Since only the fraction of DNA constituting the bridge was processed by TREX1, it could lead to clustered DNA breaks that are typical of chromothripsis. Accordingly, whole-genome sequencing of clones that have escaped from crisis have revealed potential signatures of chromothripsis, linking telomere shortening and chromothripsis.

### The Dynamic Nature of Crisis in Cancer

Replicative crisis has a profound antitumor effect. It functions by eliminating the majority of the cells that have lost tumor-suppressor pathways and bypassed senescence. While the tumor-suppressive aspect is undoubtable, several lines of evidence have pointed to telomere crisis as the origin of cellular transformation and malignancy. For instance, telomeres in cancer are usually shorter than those in corresponding normal tissues (Barthel et al. 2017). Furthermore, the transition from usual ductal hyperplasia to invasive cancer is accompanied by telomere-driven genomic aberrations (Chin et al. 2004). Moreover, molecular analysis of chronic lymphocytic leukemia (CLL) revealed signs of telomere fusions (Lin et al. 2010). Finally, mouse models have provided evidence that the genome instability arising in telomere crisis can promote tumorigenesis (Artandi et al. 2000). These observations have led to the idea that premalignant cells could experience a period of telomere crisis before they progress into malignant tumors and that crisis escape is an essential prerequisite for malignancy.

Crisis could contribute to tumorigenesis by creating a permissive environment for the acquisition of genetic and epigenetic alterations. These changes can allow a minority of cells to exit crisis and progress into full-blown invasive cancer. In line with this idea, one out of 10^5^–10^7^ cells are reported to escape from crisis and evolve progressively to a neoplastic state, potentially bypassing through the M3 plateau. Postcrisis or post-M3 cells exhibit many properties similar to those of the cells composing malignant tumors, including sustained proliferative signaling, replicative immortality, and genomic catastrophe (Counter et al. 1994, Huschtscha & Holliday 1983, Jones et al. 2014, Maciejowski et al. 2015, Montalto et al. 1999).

A long-standing controversy concerns the relevance of crisis and crisis escape to tumorigenesis in vivo. While no current specific biomarkers exist, a PCR (polymerase chain reaction)-based assay has been developed to detect telomere fusion events, a hallmark feature of crisis cells. Using this approach, researchers have observed evidence for crisis in CLL, colorectal adenomas, and other tumors (Capper et al. 2007, Jones et al. 2014, Lin et al. 2010).

## CRISIS ESCAPE THROUGH TELOMERE MAINTENANCE MECHANISM ACTIVATION

Immortalization of human cells is associated with an activation of a TMM. The majority of cancers reactivate the reverse transcriptase telomerase. This ribonucleoprotein complex uses its RNA subunit *TERC* as a template to catalytically add telomeric repeats to the 3′ end of telomeres during S phase (Wu et al. 2017). Alternatively, 5 to 15% of cancer cells use recombination-mediated alternative lengthening of telomeres (ALT) to elongate their telomeres (Dilley & Greenberg 2015). Accordingly, clones that escape from crisis display unlimited replicative potential, which is caused by some form of telomere stabilization by telomerase (Kim et al. 1994) but sometimes by alternate mechanisms (Bryan et al. 1995).

### TERT Promoter Mutations and Telomerase Reactivation

The most frequent noncoding somatic mutations in cancers are mutations in the promoter of the telomerase catalytic subunit *TERT* (TERTp), which can be found in up to 90% of glioblastomas (Barthel et al. 2017, Horn et al. 2013, Huang et al. 2013). Those mutations generate de novo ETS transcription factor binding sites and *TERT* expression (Chiba et al. 2015, Horn et al. 2013, Huang et al. 2013). Side-by-side comparison of wild-type and mutant TERTp isogenic cells identified that TERTp mutation–induced *TERT* expression was sufficient to stabilize critically short telomeres and enable crisis bypass (Chiba et al. 2017). Despite continuous growth, the TERTp mutation–induced telomerase activity failed to prevent bulk telomere shortening and consequently led to an accumulation of critically short telomeres and telomeric fusions. Thus, TERTp mutations may allow sufficient telomerase activity to limit telomere-driven genome instability to an optimal level for carcinogenesis that promotes both growth and the acquisition of further genetic alterations. The observed gradual telomerase activity upregulation and telomere length stabilization in later passages suggest that mutant TERTp cells acquired additional, currently unknown genetic or epigenetic alterations that promote telomerase activity (Chiba et al. 2017). It will be interesting to identify the nature and trigger of these events and whether they support telomere maintenance independently of TERTp mutations.

Furthermore, transcriptional upregulation of *TERT* expression has been linked to *TERT* rearrangements that frequently result in an enhancer being in proximity to the *TERT* locus and in changes in the local chromatin (Peifer et al. 2015, Valentijn et al. 2015, Zhao et al. 2009). Alternatively, *TERT* amplification (Barthel et al. 2017), as well as overexpression or amplification of cancer-driving oncogenes like *MYC*, which binds TERTp, can lead to elevated *TERT* expression (Peifer et al. 2015). *MYC* is frequently amplified on extrachromosomal DNA (Stephens et al. 2011), and these DNA species likely originate as a consequence of genomic instability during telomere-driven crisis, facilitate crisis escape, and drive tumorigenesis (Verhaak et al. 2019).

Unexpectedly, TERTp is usually methylated in *TERT*-expressing cancers, independently of ETS binding (Barthel et al. 2017, Lee et al. 2019). The exact mechanisms by which TERTp methylation promotes *TERT* expression is currently unknown; however, it has been proposed that partial TERTp methylation prevents the transcriptional repressor CTCF from binding to the TERTp and consequently counteracts *TERT* repression (Renaud et al. 2007).

Finally, a subset of *TERT-*expressing tumors show genetic *TERT* alterations that may modulate telomerase activity, whereas no *TERC* mutations and structural variants are detected (Barthel et al. 2017). Since *TERC*, in contrast to *TERT* (Kim et al. 1994), is ubiquitously expressed in all human tissues (Feng et al. 1995) and since ectopic *TERT* expression is sufficient to immortalize human primary cells, there is likely no evolutionary pressure during tumorigenesis to select for activating *TERC* mutations. Nevertheless, 4% of the investigated cancers showed *TERC* amplification, which was associated with increased telomere length (Barthel et al. 2017), resembling earlier findings that telomeres are strongly elongated by combined ectopic expression of *TERT* and *TERC* (Cristofari & Lingner 2006) and that *TERC* expression restricts telomerase activity in *TERT*-expressing human embryonic stem cells (Chiba et al. 2015).

Taken together, telomerase reactivation is the mechanism employed by most human cancers to achieve immortality (Barthel et al. 2017, Shay & Bacchetti 1997). However, *TERT* activation is likely not restricted to the abovementioned mechanisms. Future analysis may reveal additional epigenetic and posttranslational mechanisms promoting telomerase activation of cancer cells.

### Alternative Lengthening of Telomeres

In total, 5–15% of all tumors, in particular, tumors of mesenchymal origin and some brain tumors, utilize ALT to maintain their telomeres (Bryan et al. 1995, Dilley & Greenberg 2015). For this, ALT cells employ two distinct repair pathways: RAD51-dependent HR and RAD52-dependent homology-directed repair (Hoang & O’Sullivan 2020). ALT cells display characteristic nuclear ALT-associated promyelocytic leukemia (PML) bodies (APBs), which are active sites of telomeric recombination, synthesis, and generation of extrachromosomal telomeric repeats (ECTRs) (Yeager et al. 1999, Zhang et al. 2019). APBs contain clusters of telomeres; shelterin; proteins associated with DNA repair, DNA replication, and chromatin organization; and single- and double-stranded ECTRs (Zhang et al. 2019). Interestingly, ALT cells have lost expression of STING and in some cases of cGAS (Chen et al. 2017). This cytoplasmic DNA sensory pathway also recognizes ECTRs and triggers type I innate immune response (Tanaka & Chen 2012) and autophagy (Gui et al. 2019). As ECTRs are an intrinsic property of the ALT mechanism, loss of cGAS-STING may therefore be a required adaptation in the establishment of ALT.

Central for ALT are changes in telomeric chromatin and architecture to facilitate accessibility of telomeres and recombination (O’Sullivan & Almouzni 2014). Indeed, ALT telomeres accumulate telomeric variant repeats presumably due to the usage of proximal telomeric regions as recombination templates, leading to the recruitment of nuclear receptors like COUP-TF2, TR2, and TR4 and changes in telomere organization (Conomos et al. 2012, 2014; Marzec et al. 2015). Additionally, ALT telomeres are not only less compacted based on micrococcal nuclease digestion but also show changes in telomeric chromatin (Episkopou et al. 2014). The finding that depletion of the histone chaperone ASF1 induces ALT in telomerase-positive cells with long telomeres (O’Sullivan et al. 2014) highlights the critical role of telomeric chromatin for ALT. Whereas it was initially reported that ALT telomeres have reduced levels of histone H3K9me3 (Episkopou et al. 2014), a marker for heterochromatin, recent findings imply that histone methyltransferase SETDB1-dependent atypical heterochromatization of ALT telomeres is partially required for ALT (Gauchier et al. 2019). This is supported by the meta-analysis of published human chromatin immunoprecipitation sequencing data sets indicating that ALT telomeres are heterochromatic (Cubiles et al. 2018). Thus, the controversy about the telomeric chromatin state requires further investigation to finally determine the mechanistic consequences of telomeric chromatin on TMMs and telomere biology in general.

Finally, mutations in α-thalassemia/mental retardation syndrome X-linked protein (ATRX), death domain-associated protein (DAXX), and the histone variant H3.3 are frequently associated with ALT (Heaphy et al. 2011, Schwartzentruber et al. 2012). Indeed, approximately 90% of 22 well-characterized ALT cell lines are defective for ATRX (Lovejoy et al. 2012), and the ALT phenotype can be suppressed by ectopic ATRX expression (Clynes et al. 2015). Together, ATRX and DAXX form a complex that deposits histone H3.3 replication independently at pericentromeres and telomeres (Dyer et al. 2017). Additionally, ATRX contributes to unperturbed telomere DNA replication (Clynes et al. 2015, Law et al. 2010, Nguyen et al. 2017) and proper sister telomere cohesion (Lovejoy et al. 2020, Ramamoorthy & Smith 2015). The direct impact of ATRX deficiency on the establishment of ALT has not been shown until recently (Li et al. 2019). A recent study has shown that CRISPR (clustered regularly interspaced short palindromic repeats)-Cas9-generated *ATRX* knockout cells enter a premature growth plateau from which they escape by activating ALT, in contrast to control cells, which escape crisis by telomerase activation (Li et al. 2019). It will be interesting to analyze the nature of this premature growth plateau in more detail and define events required to escape from it. It remains to be tested whether STING deficiency affects the growth kinetics of *ATRX* knockout cells and thereby promotes the establishment of ALT.

Taken together, the current ALT model suggests that ALT induction is driven by altered telomeric chromatin (Gauchier et al. 2019, O’Sullivan & Almouzni 2014), which, on the one hand, increases the accessibility of telomeres for recombination and, on the other hand, facilitates telomeric replication stress. The persistent telomeric replication stress, which induces stalled replication forks, under-replicated regions, and DSBs, generates telomeric substrates for the recombination machinery in a highly recombination-prone environment, which is presumably established by the colocalization of PML bodies and telomeres in APBs. Recent evidence suggests that loss of *ATRX* triggers decompaction and alterations of telomeric chromatin (Kim et al. 2019, Li et al. 2019), as well as the increase of telomeric replication stress (Clynes et al. 2015, Law et al. 2010, Nguyen et al. 2017) and telomeric damage, thereby facilitating the progressive establishment of ALT (Li et al. 2019). This is supported by another study indicating that telomeric damage and increased recombination, which are both hallmarks of ALT, are induced in the absence of *ATRX* due to the transient loss of *macroH2A1.2* upon replication stress (Kim et al. 2019).

### When Neither Telomerase nor ALT Is Active

A considerable fraction of cancers show no apparent TMM (Barthel et al. 2017). There are at least three not mutually exclusive explanations for the lack of detectable TMM in those tumors: First, some tumors may be miscategorized as TMM-negative due to technical limitations (Barthel et al. 2017). Second, some tumors have never experienced telomere crisis and, consequently, no evolutionary pressure to activate a TMM. Early in life, telomeres are long enough to permit sufficient cell divisions for tumorigenesis. Concordantly, ~50% of neuroblastomas, a pediatric tumor of the sympathetic nervous system and the most common solid cancer in infants, show no TMM activation, frequent spontaneous regression, or differentiation and have a favorable prognosis (Peifer et al. 2015). Third, some cells acquire epigenetic or genetic alterations to escape from crisis without activating a TMM. Indeed, cells depleted for key autophagy components or the cGAS-STING pathway are capable of proliferating past crisis independently of TMMs (Nassour et al. 2019). In accordance with these observations, not all postcrisis clones appear to activate a TMM, and the escape from crisis does not always lead to immortality. Thus, TMM activation might not be an essential prerequisite for crisis escape.

## THE MULTISTEP MODEL OF CRISIS ESCAPE

**Figure 3:**
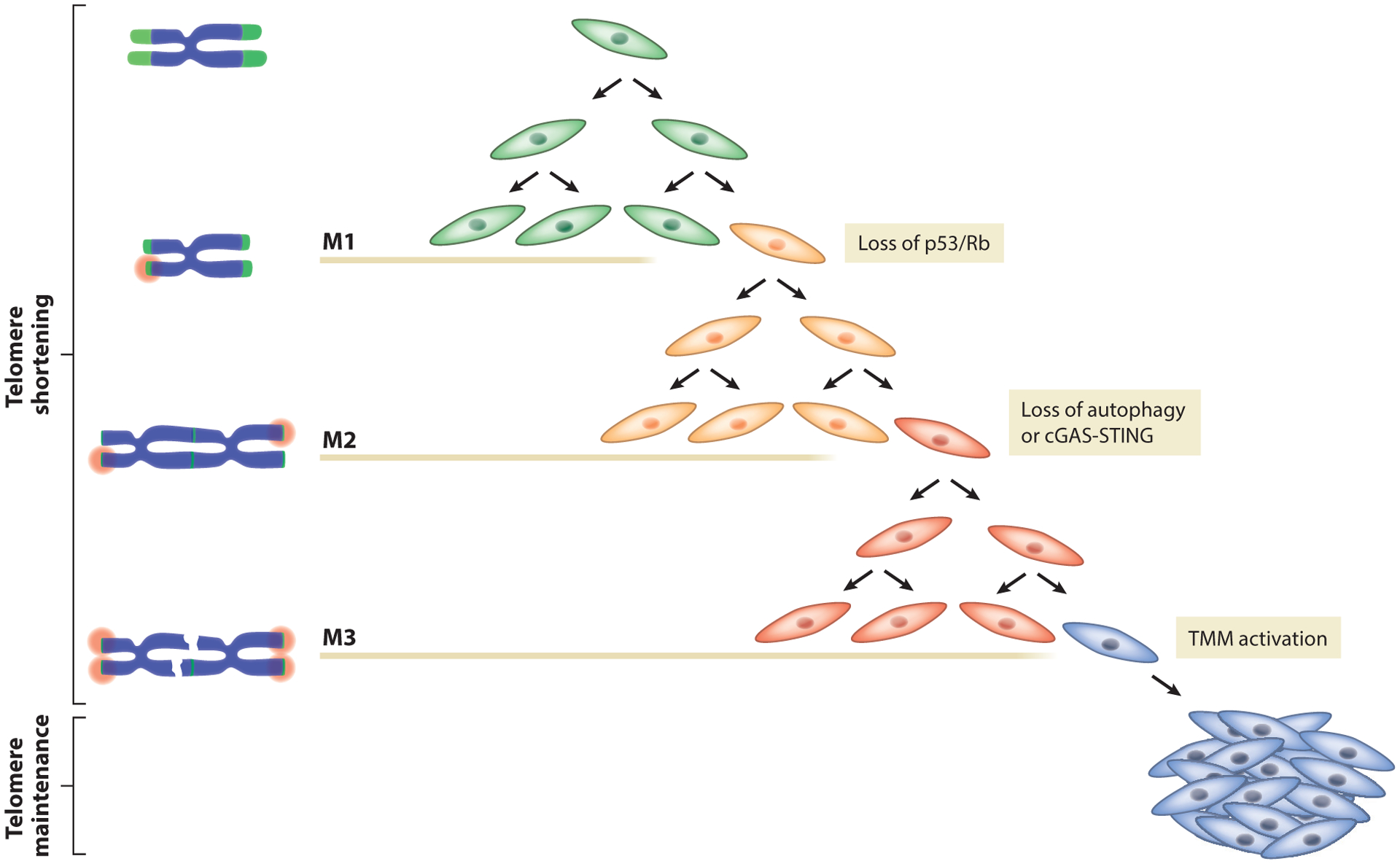
Three-stage immortalization model. Cellular immortalization requires a series of genetic and epigenetic alterations that allow cells to circumvent three proliferative barriers that restrain inappropriate cell growth. Senescence (M1) is a stable form of cell cycle arrest induced when one or a few telomeres become recognized as broken DNA ends and activate a signaling network known as DDR. Cells that gain additional oncogenic changes (p53/Rb loss) can bypass senescence and continue to divide until multiple critically shortened telomeres initiate crisis (M2), a period of increased chromosome fusions and extensive widespread cell death. Loss of the cGAS-STING/autophagy pathway allows cells to evade cell death, bypass crisis, and enter a third proliferation barrier, termed M3. Telomere dysfunction and genomic instability increase from growing cells to M3 cells, enhancing the chance of acquiring additional driver mutations and activating a TMM. M3 represents the last barrier to cancer development, and cells that escape M3 achieve proliferative immortality. Abbreviations: DDR, DNA damage response; M1/2/3, mortality stage 1/2/3; Rb, retinoblastoma protein; TMM, telomere maintenance mechanism.

The transition from normal to cancerous cells involves multiple genetic and epigenetic events, including the activation proto-oncogenes and the inactivation of tumor suppressors. These changes must occur successively through a multistep mode. Additionally, cells have to acquire alterations that enable them to overcome proliferation barriers and activate a TMM. The molecular events regulating the escape from crisis are ill defined. In the current model, spontaneous TMM activation enables some cells to exit crisis and generate immortal cell lines. This model, however, is too simplistic and does not take into account recent data on autophagy-dependent cell death during crisis (Nassour et al. 2019). Therefore, we propose a revised model of crisis escape. In addition to the known crisis escape by TMM activation, cells can also silence autophagy or the cytosolic DNA-sensing pathway to evade cell death in crisis through presently unknown mechanisms (Figure 3). These TMM-negative crisis escapees experience M3 associated with high genomic instability, enhancing the chance of activating a TMM and acquiring additional driver mutations, promoting the emergence of fully transformed cancer cells (Figure 3).
